# Human Mesenchymal Stem Cells Are Resistant to Paclitaxel by Adopting a Non-Proliferative Fibroblastic State

**DOI:** 10.1371/journal.pone.0128511

**Published:** 2015-06-01

**Authors:** Dale B. Bosco, Rachael Kenworthy, Diego A. R. Zorio, Qing-Xiang Amy Sang

**Affiliations:** 1 Institute of Molecular Biophysics, Florida State University, Tallahassee, Florida, United States of America; 2 Department of Chemistry and Biochemistry, Florida State University, Tallahassee, Florida, United States of America; Stony Brook University, UNITED STATES

## Abstract

Human mesenchymal stem cell (hMSC) resistance to the apoptotic effects of chemotherapeutic drugs has been of major interest, as these cells can confer this resistance to tumor microenvironments. However, the effects of internalized chemotherapeutics upon hMSCs remain largely unexplored. In this study, cellular viability and proliferation assays, combined with different biochemical approaches, were used to investigate the effects of Paclitaxel exposure upon hMSCs. Our results indicate that hMSCs are highly resistant to the cytotoxic effects of Paclitaxel treatment, even though there was no detectable expression of the efflux pump P-glycoprotein, the usual means by which a cell resists Paclitaxel treatment. Moreover, Paclitaxel treatment induces hMSCs to adopt a non-proliferative fibroblastic state, as evidenced by changes to morphology, cellular markers, and a reduction in differentiation potential that is not directly coupled to the cytoskeletal effects of Paclitaxel. Taken together, our results show that Paclitaxel treatment does not induce apoptosis in hMSCs, but does induce quiescence and phenotypic changes.

## Introduction

Human adult mesenchymal stem cells (hMSCs) are a class of multi-potent cells that can readily differentiate into adipocytes, chondrocytes, and osteoblasts [1]. These cells have been of particular interest over the past decade due to their tissue regenerative potential. However, investigations have recently turned toward understanding the role hMSCs play in the development and progression of cancer. Tumor microenvironments often produce a number of chemokines (*e*.*g*., CCL25, TGF-β) that can attract circulating hMSCs [2–3]. Once inside the tumor, hMSCs attempt to “repair” these microenvironments, since they appear damaged in comparison to the surrounding normal tissue [4–5]. Concurrent with these activities, numerous cross-talk interactions are thought to occur between tumor and stem cells, which can lead to cancer progression. Current literature has implicated hMSCs in the promotion of osteosarcoma and colorectal cancer, metastasis of breast cancers, as well as chemotherapeutic resistance [6–11].

One of the major hurdles in cancer treatment is the development of chemo-resistant cells within the tumor microenvironment, a trait known as multi-drug resistance (MDR) [12]. While the vast majority of research into chemotherapeutic resistance has focused on the cancer cells themselves, new research indicates that other cell types, particularly hMSCs, can act as “co-conspirators” within the tumor microenvironment, protecting cancer cells from treatment [13–14]. Mesenchymal stem cells are inherently resistant to chemotherapeutics and can confer this resistance to a number of cancer cell types, including squamous cell carcinoma and triple negative breast cancer [15–16]. A number of stem-cell-produced extracellular factors have been identified as having possible roles in promoting cancer cell chemotherapeutic resistance (*e*.*g*., Interleukin-8, platinum-induced fatty acids) [11, 16–17]. However, little has been done to investigate the effects internalized chemotherapy drugs have upon hMSCs.

Paclitaxel is one of the most widely administered anticancer agents, and operates by binding to and stabilizing microtubules [18]. Paclitaxel treatment often leads to mitotic arrest, prevention of cell division, and eventually apoptosis [19]. In this study, we investigated the effects of Paclitaxel treatment upon hMSCs. We show that Paclitaxel does inhibit hMSC proliferation, but does not induce apoptosis. We also show that Paclitaxel treatment induces hMSCs to adopt a fibroblast-like phenotype, as evidenced by changes to distinguishing markers and morphology. Finally, we show that hMSCs do not produce detectable amounts of the ATP binding cassette (ABC) transmembrane pump P-glycoprotein, which has been previously suggested to be the source of Paclitaxel resistance in cancer cells by pumping out Paclitaxel [20]. As a result, it is proposed that apoptotic resistance may instead be related to cell cycle regulation.

## Materials and Methods

### Cell culture

Low passage human mesenchymal stem cells (hMSCs) were obtained from the Tulane Center for Gene Therapy (New Orleans, LA) and cultured in α-modified minimum essential medium (αMEM, Sigma-Aldrich, St. Louis, MO) supplemented with an L-glutamine/penicillin/streptomycin cocktail (Sigma) and 20% fetal bovine serum (FBS, GE Healthcare, Piscataway, NJ). Cells were plated for 24 hrs of recovery and maintained at 37°C in a humidified atmosphere containing 5% CO_2_. Cells were then trypsinized and seeded into either 6-well plates, 96-well plates, 10 cm dishes, or two-chamber glass slides depending on experiment. All experiments were initiated after reaching 70–80% confluence unless otherwise stated.

### MTT cell proliferation assay

Half-maximal inhibitory concentration (IC_50_) was determined using the metabolic dimethyl thiazolyl diphenyl tetrazolium salt (MTT) assay, which measures conversion of MTT into formazan by mitochondrial succinate dehydrogenase and other oxidoreductases. 500 cells/well were seeded into 96-well plates and allowed to reach 70–80% confluence. Serial dilutions of Paclitaxel, synthesized by Dr. Robert A. Holton’s laboratory (Florida State University, Tallahassee, FL) [21–22], were then used to treat cells, with final concentrations ranging from 30–250,000 nM. After 72 hrs, MTT (M5655, Sigma) was added for a final concentration of 1 mg/mL. Plates were incubated for 60 mins and then centrifuged. Supernatant was removed and 200 μL DMSO added to each well. After 6 mins of shaking, absorbance was measured in a Synergy HT plate reader (Biotek, Winooski, VT) at 600 nm.

### Growth curve

6-well plates were seeded with 10,000 cells/well. After 24 hrs, cells were washed with phosphate buffered saline (PBS) and incubated for 24 hrs in FBS-free αMEM. Next, cells were incubated with complete αMEM for 24 hrs, then 0 hour plates were collected, fixed with 10% neutral buffered formalin for 1 hr, and stained with 5 μg/mL Hoechst 33342 (Sigma) for 5 mins. Remaining plates were then treated with either 0, 10, or 10,000 nM Paclitaxel. Plates were then collected, fixed, and stained every 24 hrs. After every time point was collected, plates were imaged with an epifluorescence capable Nikon TE2000-E2 Eclipse microscope. Quantification of stained nuclei per image with ImageJ (version 1.46c, National Institutes of Health, Bethesda, MD) was used to determine cell number and growth rates.

### Viability assay

Human MSCs in 6-well plates were treated with media supplemented with Paclitaxel, 0–100,000 nM final concentration, and incubated for 72 hrs. Cells were then trypsinized, pelleted, and resuspended into 2 mL of αMEM. Samples of each suspension were diluted 1:1 with Trypan Blue (T8154, Sigma). Live (unstained) and dead (stained) cells were counted with a hemocytometer. To determine viability, live cell counts were divided by total cell counts for each condition.

### Western blot

Human MSCs in 10 cm dishes were treated with 10 nM Paclitaxel for 12 days. Samples were collected every 72 hrs, trypsin treated, washed with cold PBS, and then pelleted via centrifugation. Protein lysates were generated using EDTA buffer (62.5 mM Tris-HCl pH 6.8, 2% SDS, 10% glycerol, 5% β-ME, 10 mM EDTA). Protein lysates were resolved in NuPAGE 4–12% Bis-Tris Gels (Life Technologies, Carlsbad, CA), transferred overnight onto PDVF membranes and probed against P-glycoprotein (SC-13131, Santa Cruz Biotechnology, Santa Cruz, CA). β-actin (A5316, Sigma) was used as a loading control. Cell lysate from the colorectal adenocarcinoma HTC-15 cell-line (CCL-225, ATCC, Manassas, VA) was generated similarly and used as a positive control.

### Quantitative real-time PCR

Human MSCs in 10 cm dishes were treated with 10 nM Paclitaxel for 12 days with cells being collected on days 0, 6, and 12. Total RNA from each time point was isolated using RNA-Bee solvent (TEL-TEST Inc., Friendswood, TX) and the miRNeasy extraction kit (Qiagen, Valencia, CA). RNA was then re-suspended in nuclease free water, with concentrations being determined with a Nanodrop UV-Vis Spectrophotometer (ThermoFisher Scientific, Waltham, MA). Integrity was measured using a 2100 Bionanlyzer (Agilent Technologies Inc. Santa Clara, CA). 1 μg of RNA was used for cDNA synthesis using qScript cDNA SuperMix (Quanta Bioscience, Gaitherburg, MD). 100 ng/μL cDNA was used as template for quantitative PCR on a 7500 Fast Real Time PCR system (Life Technologies) using PerfeCTa SYBR Green SuperMix, Low Rox (Quanta Bioscience). Relative gene expression was determined via the 2^^(-ΔΔCt)^ method [23]. Primers were purchased from Integrated DNA Technologies (IDT, Coralville, IA) (Table 1).

**Table 1:** Primer list for real-time PCR.

Gene	Forward Primer	Reverse Primer
**CD9**	5’-GGGGGCGTGGAACAGTTTAT-3’	5’-GCGCCGATGATGTGGAATTT-3’
**CD106**	5’-TTCTGTGCCCACAGTAAGGC-3’	5’-GCTGGAACAGGTCATGGTCA-3’
**CD146**	5’-TGTGTAGGGAGGAACGGGTA-3’	5’-CAAACCACTCGACTCCACAGT-3’
**CD166**	5’-TTCTGCCTCTTGATCTCCGC-3’	5’-AGGTACGTCAAGTCGGCAAG-3’
**GAS1**	5’-CCTCATTCAGCTCAACCACA-3’	5’-CTTGGTGGACTTGCAGTTCT-3’
**ITGA11**	5’-CGGCCTCCAGTATTTTGGCT-3’	5’-GGAGGCTGGCATTGATCTGA-3’
**MMP-1**	5’-GGCCACAAAGTTGATGCAGTT-3	5’-TTCCTGCAGTTGAACCAGCTA-3’
**MMP-3**	5’-CCATCTCTTCCTTCAGGCGT-3’	5’-ATGCCTCTTGGGTATCCAGC-3’
**β-ACTIN**	5’-AGTCCTGTGGCATCCACGAAACTA-3’	5’-ACTCCTGCTTGCTGATCCACATCT-3’

### Adipogenic differentiation

Human MSCs in Lab-Tek II 2-chamber culture slides (Nalgen Nunc, Rochester, NY) were induced to undergo adipogenesis in the presence of either 0, 10, or 10,000 nM Paclitaxel via the addition of 0.5 μM dexamethasone (Sigma), 0.5 μM isobutylmethylxanthine (IBMX; Sigma), and 50 μM indomethacin (Sigma) to the culture medium as described [24]. After 21 days of differentiation, hMSCs were washed with PBS and fixed with neutral buffered formalin for 1 hr. After fixation, cells were stained with 0.1 μg/mL Nile Red (Sigma) for 5 mins, washed with PBS, stained with 5 μg/mL Hoechst 33342 (Sigma) for 5 mins, washed again, and finally mounted with the SlowFade antifade reagent kit (Life Technologies). Cells were imaged via fluorescent microscopy and relative adipogenesis was determined with ImageJ by standardizing area of staining in each image to respective nuclei number and then normalizing against respective controls.

### Immunocytochemistry

Microtubule bundling analysis via fluorescent microscopy was performed on hMSCs grown in 2-chamber culture slides and induced to undergo adipogenesis in the presence of either 0, 10, or 10,000 nM Paclitaxel. After 21 days, cells were fixed in PBS containing 10% formaldehyde and 3% sucrose for 10 mins. Cells were then washed with PBS and permeabilized with 2% Triton-X 100 in PBS for 5 mins. Cells were incubated with 1:1000 mouse anti-α tubulin antibody (T5168, Sigma) for 60 mins at 37°C, washed with PBS, incubated with 1:1000 fluorescein isothiocyanate (FITC) conjugated goat anti-mouse antibody (F4018, Sigma) for 60 mins at 37°C, then washed again with PBS. Finally, slides were mounted after nuclei staining and imaged via fluorescent microscopy.

### Statistical analysis

Experiments described above were performed with at least three independent samples per data point. MTT, qRT-PCR, proliferation, and viability results are expressed as the mean ± standard deviation. Differentiation results are expressed as the mean ± standard error. Statistical analyses were performed with the least significant difference correction for one-way analysis of variance (ANOVA) for multiple comparisons, with statistically significant values defined as *P* < 0.05.

## Results

### Effect of Paclitaxel treatment upon proliferation and viability

**Fig 1:**
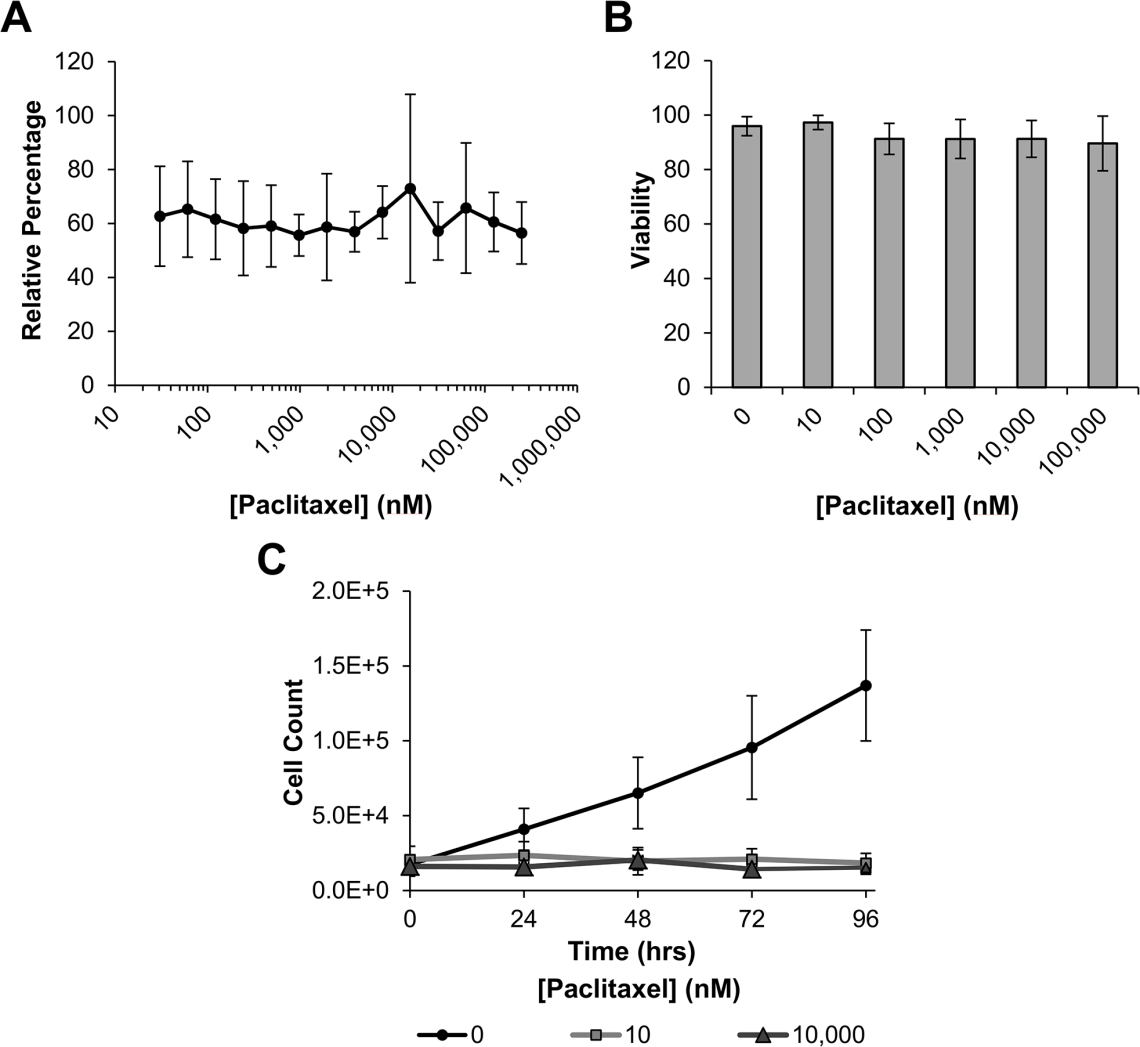
Proliferation and viability of hMSCs in the presence of Paclitaxel. (A) Human MSCs were incubated with 30–250,000 nM Paclitaxel for 72 hrs, then treated with dimethyl thiazolyl diphenyl tetrazolium salt (MTT) and processed. Absorbance was measured at 600 nM and compared with controls to produce relative values. (B) Human MSCs were incubated for 72 hrs with Paclitaxel at various concentrations. After incubation, cells were counted via hemocytometer and compared with controls to determine relative viability. After 72 hrs viability was determined to be above 90% in hMSCs treated with up to 100,000 nM Paclitaxel. (C) Growth curves were generated by counting stained nuclei of hMSCs treated with either 0, 10, or 10,000 nM Paclitaxel for various lengths of time. It was revealed that Paclitaxel at both 10 and 10,000 nM completely inhibited proliferation of hMSCs. Results for each experiment are displayed as mean ± standard deviation.

To examine the effects Paclitaxel treatment has upon human mesenchymal stem cell (hMSC) proliferation and viability, we used the metabolic dimethyl thiazolyl diphenyl tetrazolium salt (MTT) assay. Since it has been described that hMSCs are relatively resistant to Paclitaxel [11], cells were treated with a broad panel of Paclitaxel concentrations (30–250,000 nM) for 72 hrs, then treated with MTT and processed. Cell number was quantified by measuring absorbance at 600 nM. Fig 1A shows that upon treatment there was a uniform reduction in cell number when compared to control groups, even though there was no appreciable difference between treatment concentrations.

**Fig 2:**
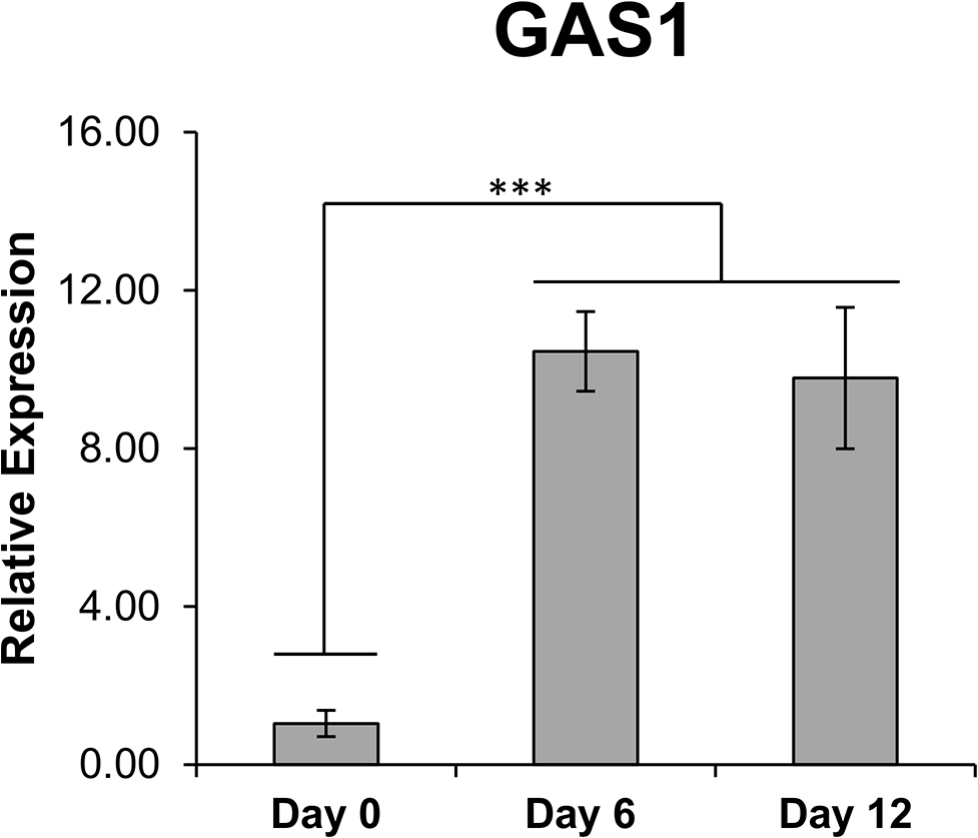
Quantitative real-time PCR of growth arrest specific factor 1 (GAS1) in hMSCs treated with Paclitaxel over time. Human MSCs were treated with 10 nM Paclitaxel for up to 12 days, with samples being taken at various time points. Relative expression for each gene was calculated via the 2^^(-ΔΔCt)^ method with Day 0 expression acting as the basis for comparison. Results are displayed as mean ± standard deviation. *** P < 0.01

To determine if the observed reduction was due to decreases in viability or proliferation, Trypan Blue exclusion and growth curve assays were performed. After 72 hrs of Paclitaxel treatment, no perceived difference in viability was observed up to 100,000 nM (Fig 1B). However, when treated with either 10 or 10,000 nM Paclitaxel, there was a complete abatement in cellular proliferation when compared to controls (Fig 1C). To investigate if the lack of proliferation was due to the cells becoming quiescent we examined expression of growth arrest specific factor 1 (GAS1). GAS1 is a key regulator of the cell cycle which halts division by blocking entry into S phase, inducing quiescence [25]. Cells were treated with the more physiologically relevant concentration of 10 nM Paclitaxel for 12 days with samples being collected at various time points. Quantitative RT-PCR was used to determine the change in GAS1 expression over time in response to Paclitaxel treatment. Fig 2 shows that upon treatment, there is a significant increase in GAS1 expression coinciding with the secession of proliferation, indicating that treated cells are becoming quiescent. These results indicate that the hMSCs are highly resistant to the apoptotic effects of paclitaxel treatment, even though there is a clear effect upon proliferation.

### Adoption of fibroblast-like characteristics

**Fig 3:**
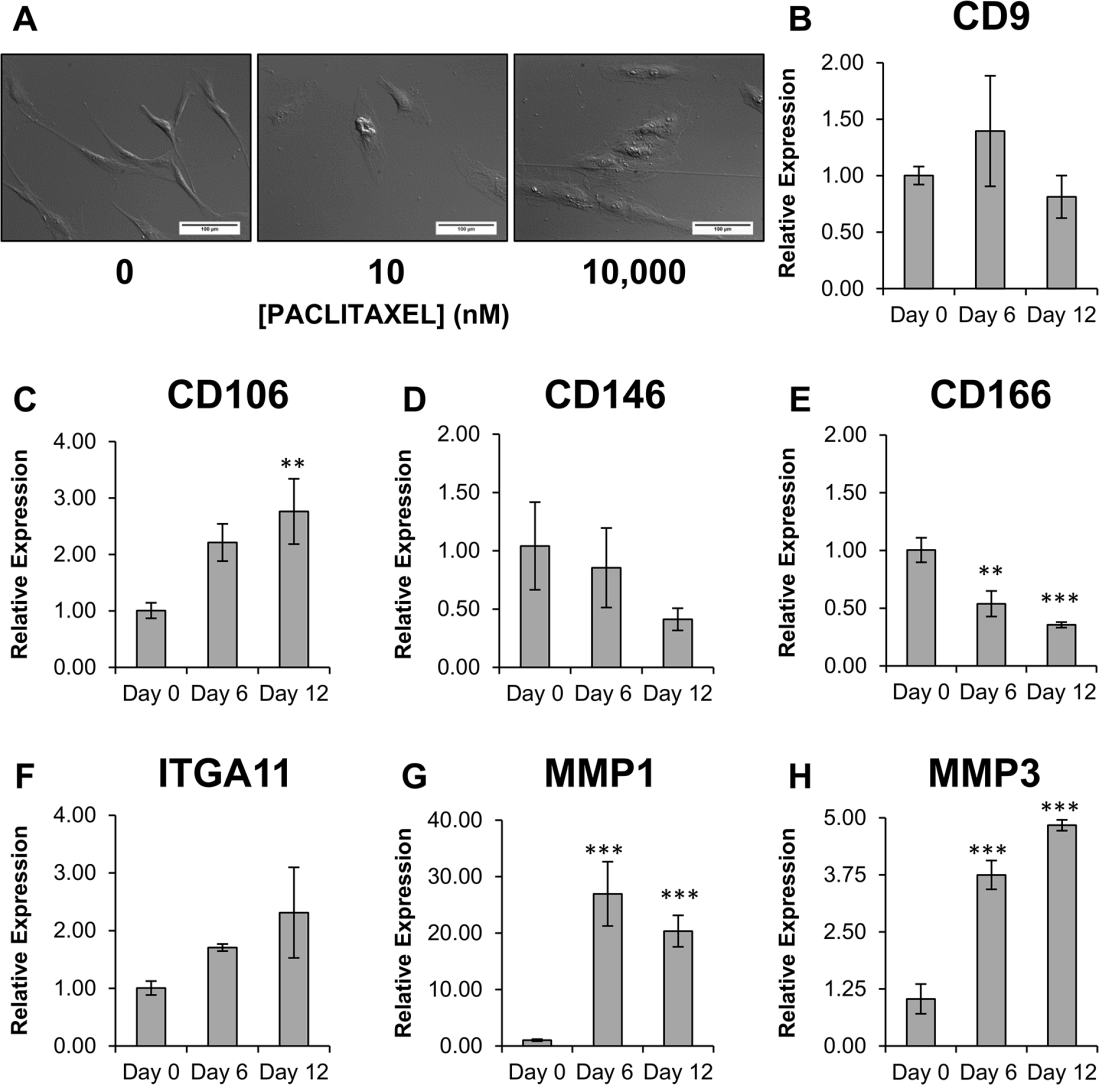
Characterization of the fibroblast-like state hMSCs adopt when treated with Paclitaxel. (A) After 72hrs of exposure to either 10 or 10,000 nM Paclitaxel hMSCs transition from the typical spindle-shaped morphology, seen in the untreated hMSCs, to one that is more broad and flat. Quantitative Real-Time PCR was used to determine expression levels of (B) CD9 (C) CD106 (D) CD146 (E) CD166 (F) integrin alpha 11 (ITGA11) (G) matrix metalloproteinase 1 (MMP-1) and (H) MMP-3 in hMSCs treated with 10 nM Paclitaxel over time. Results are displayed as mean ± standard deviation. ** P < 0.05, *** P < 0.01. Scale bar (A) 150 μm.

Coinciding with the cessation of proliferation, there was a notable change to the morphology of hMSCs treated with Paclitaxel (Fig 3A). Mesenchymal stem cells normally adopt a long spindle shape morphology when grown *in vitro*, however, within as little as 24 hrs after Paclitaxel treatment, cells began to adopt a flat, broad fibroblastic appearance. Consequently, we wanted to examine if Paclitaxel treatment was inducing the hMSCs to adopt a fibroblast phenotype. Based on the studies by Ishii *et al*. [26], and Halfon *et al*. [27], we investigated how Paclitaxel treatment modulated the expression of the fibroblast markers matrix metalloproteinase-1 (MMP-1), MMP-3, and CD9, and the hMSC markers integrin α11 (ITGA11), CD106, CD146, and CD166.

Human mesenchymal stem cells were treated with 10 nM Paclitaxel and expression levels of the target genes were measured on day 0, 6, and 12. The most noticeable change in expression occurred with MMP-1 and MMP-3, where both showed substantial increases in expression upon Paclitaxel treatment. CD106 also showed an increase in expression on day 12 of treatment. CD166 showed a 2-fold reduction in expression upon treatment with Paclitaxel. As for CD9, CD146, and ITGA11, expression differences were observed over the course of treatment, however not to a statistically significant degree (Fig 3B–3H).

### Adipogenic differentiation in the presence of Paclitaxel

**Fig 4:**
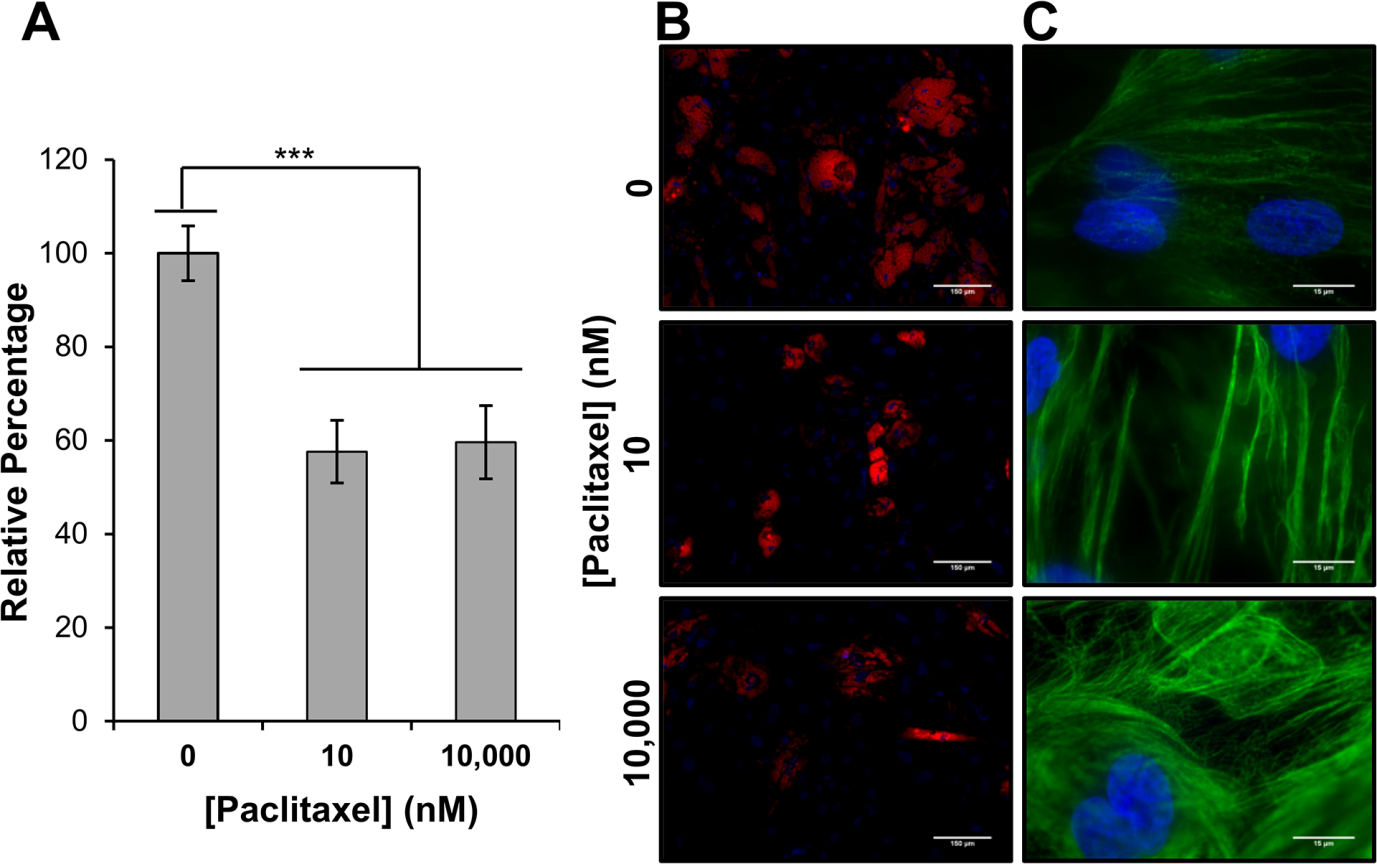
Effect of Paclitaxel upon the differentiation ability of hMSCs. (A) and (B) After 21 days of induced adipogenic differentiation in the presence of either 0, 10, or 10,000 nM Paclitaxel, cells were fixed and stained with the lipophilic dye Nile Red and Hoechst 33342. Image samples were taken of each culture and area of staining was standardized to nuclei counts for that area. A decrease in lipid accumulation of approximately 40% when compared to control was observed in hMSCs treated with either 10 or 10,000 nM Paclitaxel. (C) Microtubule staining via immunocytochemistry reveled an apparent dose-dependence upon cytoskeletal organization in the differentiated hMSCs. At 10 nM characteristic bundling was observed. At 10,000 nM over-accumulation of microtubules was observed. Results are displayed as mean ± standard error. *** P < 0.01. Scale bar (B) 150 μm, (C) 15 μm.

Given the apparent effects Paclitaxel has upon hMSC proliferation and phenotype, we investigated what effect Paclitaxel treatment had upon the differentiation capacity of hMSCs. Mesenchymal stem cells were induced toward an adipogenic lineage for 21 days via the addition of dexamethasone, isobutylmethylxanthine (IBMX), and indomethacin to the culture medium, in the presence of either 0, 10, or 10,000 nM Paclitaxel. 10 and 10,000 nM were used to determine the extent that concentration affects adipogenesis. After 21 days, cells were stained with the lipophilic dye Nile Red. Area of staining, standardized to nuclei number, was used as a measure of adipogenesis (Fig 4A and 4B). Our results show that when compared to cells treated with 0 nM Paclitaxel, there was about a 40% reduction in relative lipid accumulation in cells treated with either 10 or 10,000 nM Paclitaxel. Even though there was no perceived difference in lipid accumulation between treatment conditions, examination of hMSC microtubule structure revealed that cells were affected in a dose-dependent manner (Fig 4C). At 10 nM Paclitaxel, microtubule bundling was observed, with increases in microtubule mass being observed at 10,000 nM Paclitaxel, hallmarks of Paclitaxel dose response [28]. Consequently, it appears that Paclitaxel’s effect upon adipogenesis is not directly related to its effect upon microtubule stabilization.

### Paclitaxel-binding transmembrane transporter P-glycoprotein is not expressed in hMSCs treated with Paclitaxel

**Fig 5:**
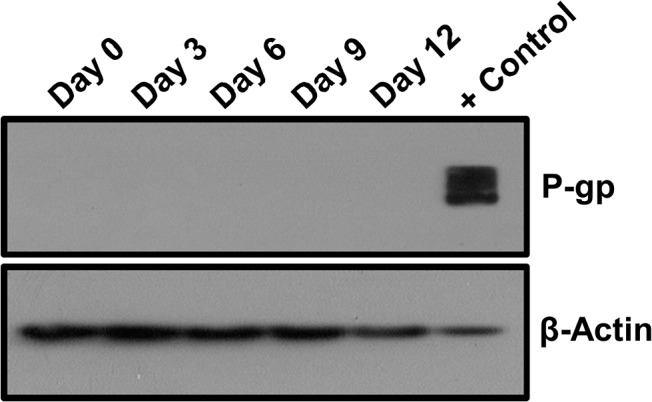
Western blot analysis of P-glycoprotein in hMSCs treated with 10nM Paclitaxel over time. Results show that the hMSCs do not natively express P-glycoprotein (P-gp), nor is expression induced by exposure to Paclitaxel. Positive control is cell lysate from the human colorectal adenocarcinoma cell line HCT-15, which constitutively expresses high levels of P-gp. β-actin was used as a loading control.

The predominate means by which cancer cells resist Paclitaxel exposure is via up-regulation of the transmembrane transport protein ATP binding cassette B1 (ABCB1), also known as P-glycoprotein 1 (P-gp) [29]. To determine if mesenchymal stem cells also produced P-gp during Paclitaxel exposure, mesenchymal stem cells were treated with 10 nM Paclitaxel for 12 days with samples being collected every 72 hrs. Western blots were probed against P-gp (Fig 5). No P-glycoprotein expression was detected at any time point during Paclitaxel treatment, indicating that hMSCs do not natively express P-gp, nor does treatment induce P-gp expression.

## Discussion

Paclitaxel is a potent chemotherapeutic that operates as a microtubule stabilizing agent, preventing GDP-induced de-polymerization of microtubule structures [18]. This in turn reduces microtubule dynamics, inducing mitotic arrest and eventually apoptosis [19]. While the exact mechanism of apoptotic induction is still unknown, evidence suggests that chromosome missegregation may be involved [30]. Regardless, mesenchymal stem cells have been shown to be highly resistant to the cytotoxic effects of Paclitaxel and other chemotherapeutic agents [31–32] and can confer this resistance to tumor microenvironments [3, 17]. While a major focus has been identifying extracellular factors relating to the conferring of resistance to the tumor microenvironment, hMSCs still internalize Paclitaxel upon treatment, as the lipophilic nature of the compound allows for easy diffusion across a cell membrane [33–34]. Consequently, we investigated the effects exposure to Paclitaxel had upon the intracellular function and viability of hMSCs.

Our first goal was to determine the concentration of Paclitaxel needed to induce apoptosis within an hMSC. MTT assays were conducted, but no perceivable effective concentration could be determined. Consequently, we wanted to deduce if the observed uniform decrease in cell number was due to a decrease in viability or a decrease in proliferative rate. We found that Paclitaxel had only a minimal effect upon viability, even though both 10 and 10,000 nM Paclitaxel completely inhibited hMSC proliferation. We also found that expression of growth arrest specific factor 1 (GAS1), which can induce cells to enter G0 phase and become quiescent [25], was up-regulated in treated hMSCs. Thus, it seems that the reductions in cell number observed in the MTT assay were due to cessation of proliferation via induction of quiescence in the treated cells, and that hMSCs are extremely resistant to the cytotoxic effects of Paclitaxel [35].

Since it was observed that treatment with Paclitaxel prompted the hMSCs to transition from their typical long spindle shape to a broader fibroblastic appearance, we decided to determine if Paclitaxel treatment was not only preventing proliferation, but also inducing the hMSCs to become fibroblastic. Distinguishing hMSCs from fibroblasts has been one of the more difficult aspects of stem cell research. Both cell types are plastic-adherent, share many of the same CD markers, and fibroblasts have a limited ability to differentiate [36]. However, recent studies have identified a number of possible markers that can be used to distinguish the two cell types: matrix metalloproteinase-1 (MMP-1), MMP-3, integrin α11 (ITGA11), CD9, CD106, CD146, and CD166 [26–27]. These markers were chosen because they were described as having a large difference in expression when comparing hMSCs to fibroblasts. We found that MMP-1, MMP-3, and CD106 all showed increased expression upon treatment with Paclitaxel, while CD166 expression was reduced upon treatment. Change in expression of CD9, CD146, and ITGA11 was not statistically significant. Increases of MMP-1 and MMP-3, and decreases of CD166 are consistent with the notion that the hMSCs are becoming fibroblastic [27]. The increase in CD106 expression and the lack of relevant change to CD9, CD146, and ITGA11 may be due to this investigation utilizing hMSCs induced towards a fibroblastic state, rather than lung or dermal derived fibroblasts as described in other studies [26–27]. Our studies do indicate, however, that Paclitaxel treatment seems to be inducing a phenotypic change within the hMSCs.

One of the most important abilities of stem cells is the capacity to differentiate into other cell types. Since Paclitaxel does not appear to affect hMSC viability, we wanted to determine what effect Paclitaxel treatment had upon the differentiation of hMSCs. Since adipogenic differentiation of hMSCs is not dependent upon proliferation [37], it was chosen to be the target lineage. To our surprise there was no Paclitaxel concentration dependence when it came to lipid accumulation, even though there was a clear effect upon the microtubule organization at the different Paclitaxel concentrations. At 10 nM, microtubule bundling was observed with over-accumulation of microtubules being seen at 10,000 nM, clear indicators of a Paclitaxel dose response [28]. Considering that treated hMSCs adopt a fibroblastic-like morphology independent of Paclitaxel concentration, it is possible that the reduced differentiation potential is due to phenotypic changes rather than cytoskeletal effects. This would provide further evidence that the hMSCs are becoming fibroblastic, since fibroblast populations do possess the ability to differentiate, but this ability is reduced when compared to hMSCs and is lost completely as the fibroblast cell populations mature [36, 38].

Since Paclitaxel resistance in cancer cells is primarily attributed to its removal from cells by the transmembrane pump P-glycoprotein (P-gp), we wanted to determine if P-gp was also involved in the promotion of hMSC Paclitaxel resistance. Normally, hMSCs do not express P-gp [39], even though reports have indicated that hMSCs can remove internalized paclitaxel [33,35] to the point that hMSCs have been investigated for their ability to deliver and release paclitaxel into tumor sites [35, 40–42]. We showed, via Western blot analysis, that untreated hMSCs do not express P-gp protein and that the presence of Paclitaxel did not induce its expression. This is in contrast to cancer stem cells, which constitutively express high levels of P-gp [43]. Moreover, the lack of induced P-gp expression is curious as simple Paclitaxel conditioning experiments have generated several resistant cell lines that produce high levels of P-gp from sensitive parental lines that produce little to no P-gp [44–45]. These experiments often require no more than culturing cells in stepwise increasing Paclitaxel concentrations. Thus, hMSC resistance to Paclitaxel does not appear to depend on P-gp expression, providing evidence that hMSCs possess alternative means of resistance.

**Fig 6:**
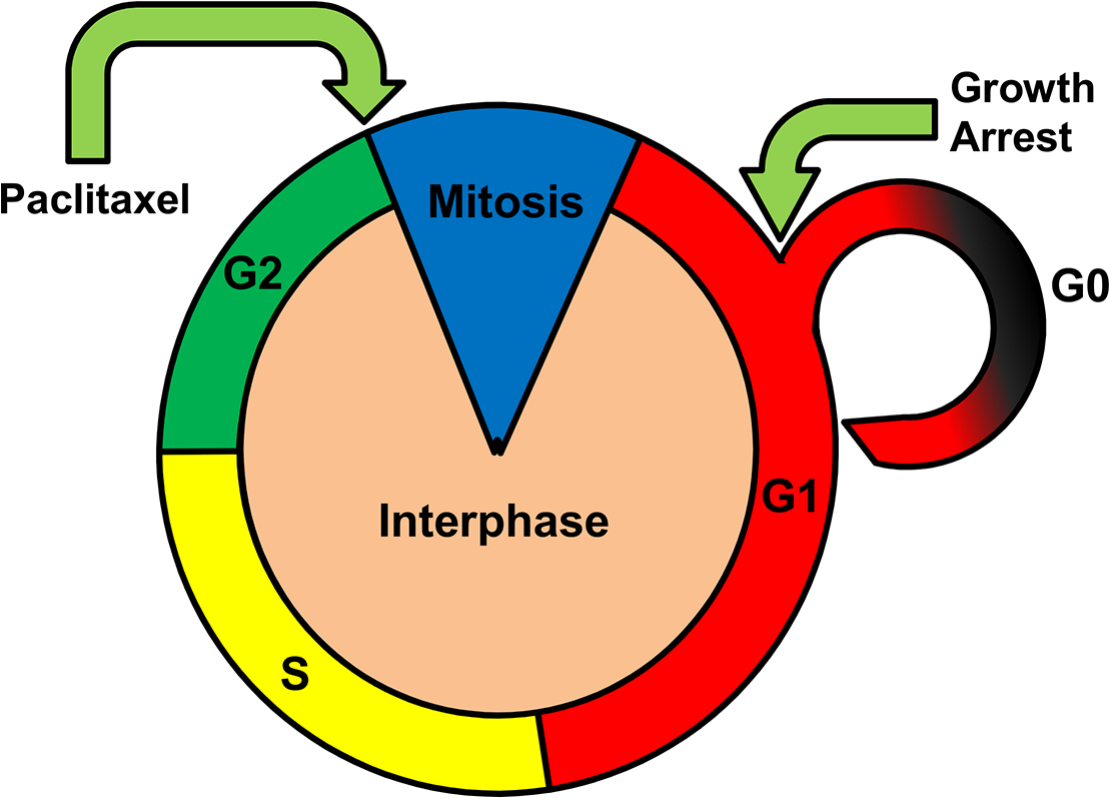
Cell cycle regulation may provide Paclitaxel resistance in hMSCs. By inducing cells to enter quiescence before the G2/M transition needed for Paclitaxel action, it is possible that hMSCs utilize cell cycle regulation to protect themselves from the cytotoxic effects of Paclitaxel.

Given the apparent induction of quiescence in hMSCs upon Paclitaxel treatment evidenced by cessation of proliferation and increased GAS-1 expression, and the fact that procession through M phase of the cell cycle is critical for Paclitaxel efficacy [19,46], it is possible that the hMSCs are utilizing cell cycle regulation to escape the apoptotic effects of Paclitaxel (Fig 6). Mesenchymal stem cells are normally long-lived cells that can readily enter and exit quiescence depending on external signals [47]. Moreover, blockage of the cell cycle at either G1 or G2 has been shown to greatly reduce the apoptotic effects of Paclitaxel in cancer cells [48]. Cancer sub-populations have also been shown to evade the effects of Fluorouracil (5FU) by entering a similar state of quiescence [49]. Therefore, the ability to regulate the cell cycle may be a factor in hMSC resistance to Paclitaxel.

## Conclusion

In this study we investigated the effects of Paclitaxel treatment on human mesenchymal stem cells (hMSCs). We found that Paclitaxel abolished hMSC proliferation without affecting viability and that Paclitaxel resistance may be due to regulation of the cell cycle and not the expression of P-glycoprotein (P-gp). Additionally, we observed that Paclitaxel treatment may induce hMSCs to adopt a fibroblastic-like phenotype based on changes to expression of distinguishing gene markers, morphology, and a reduction in differentiation potential that does not appear to be directly coupled to cytoskeletal effects of Paclitaxel.

## References

[1] Pittenger MF, Mackay AM, Beck SC, Jaiswal RK, Douglas R, Mosca JD, et al Multilineage potential of adult human mesenchymal stem cells. Science. 1999 4 2; 284(5411): 143–147. 10102814 10.1126/science.284.5411.143

[2] Xu S, Menu E, De Becker A, Van Camp B, Vanderkerken K, Van Riet I. Bone marrow-derived mesenchymal stromal cells are attracted by multiple myeloma cell-produced chemokine CCL25 and favor myeloma cell growth in vitro and in vivo. Stem Cells. 2012 2; 30(2): 266–279. 10.1002/stem.787 22102554

[3] Shinojima N, Hossain A, Takezaki T, Fueyo J, Gumin J, Gao F, et al TGF-beta mediates homing of bone marrow-derived human mesenchymal stem cells to glioma stem cells. Cancer Res. 2013 4 1; 73(7): 2333–2344. 10.1158/0008-5472.CAN-12-3086 23365134 PMC3644948

[4] Dittmer J. Mesenchymal stem cells: "repair cells" that serve wounds and cancer? ScientificWorldJournal. 2010 6 29; 10: 1234–1238. 10.1100/tsw.2010.119 20602080 PMC5763672

[5] Yang X, Hou J, Han Z, Wang Y, Hao C, Wei L, et al One cell, multiple roles: Contribution of mesenchymal stem cells to tumor development in tumor microenvironment. Cell Biosci. 2013 1 21; 3(1): 5-3701-3-5. 10.1186/2045-3701-3-5 PMC3693909 23336752

[6] Hurst DR, Welch DR. A MSC-ing link in metastasis? Nat Med. 2007 11; 13(11): 1289–1291. 17987025 10.1038/nm1107-1289 PMC2267025

[7] Karnoub AE, Dash AB, Vo AP, Sullivan A, Brooks MW, Bell GW, et al Mesenchymal stem cells within tumour stroma promote breast cancer metastasis. Nature. 2007 10 4; 449(7162): 557–563. 17914389 10.1038/nature06188

[8] Bian ZY, Fan QM, Li G, Xu WT, Tang TT. Human mesenchymal stem cells promote growth of osteosarcoma: Involvement of interleukin-6 in the interaction between human mesenchymal stem cells and saos-2. Cancer Sci. 2010 12; 101(12): 2554–2560. 10.1111/j.1349-7006.2010.01731.x 20874851 PMC11159660

[9] Chaturvedi P, Gilkes DM, Wong CC, Kshitiz, Luo W, Zhang H, et al Hypoxia-inducible factor-dependent breast cancer-mesenchymal stem cell bidirectional signaling promotes metastasis. J Clin Invest. 2013 1 2; 123(1): 189–205. 10.1172/JCI64993 23318994 PMC3533298

[10] De Boeck A, Pauwels P, Hensen K, Rummens JL, Westbroek W, Hendrix A, et al Bone marrow-derived mesenchymal stem cells promote colorectal cancer progression through paracrine neuregulin 1/HER3 signalling. Gut. 2013 4; 62(4): 550–560. 10.1136/gutjnl-2011-301393 22535374

[11] Houthuijzen JM, Daenen LG, Roodhart JM, Voest EE. The role of mesenchymal stem cells in anti-cancer drug resistance and tumour progression. Br J Cancer. 2012 6 5; 106(12): 1901–1906. 10.1038/bjc.2012.201 22596239 PMC3388567

[12] Gottesman MM, Fojo T, Bates SE. Multidrug resistance in cancer: role of ATP-Dependent Transporters. Nature Reviews Cancer. 2002 1, 2(1): 48–58 11902585 10.1038/nrc706

[13] Spaeth EL, Dembinski JL, Sasser AK, Watson K, Klopp A, Hall B, et al Mesenchymal stem cell transition to tumor-associated fibroblasts contributes to fibrovascular network expansion and tumor progression. PLoS One. 2009; 4(4): e4992 10.1371/journal.pone.0004992 19352430 PMC2661372

[14] Bergfeld SA, Blavier L, DeClerck YA. Bone marrow-derived mesenchymal stromal cells promote survival and drug resistance in tumor cells. Mol Cancer Ther. 2014 4; 13(4): 962–975. 10.1158/1535-7163.MCT-13-0400 24502925 PMC4000239

[15] Scherzed A, Hackenberg S, Froelich K, Kessler M, Koehler C, Hagen R, et al BMSC enhance the survival of paclitaxel treated squamous cell carcinoma cells in vitro. Cancer Biol Ther. 2011 2 1; 11(3): 349–357. 21127403 10.4161/cbt.11.3.14179

[16] Chen DR, Lu DY, Lin HY, Yeh WL. Mesenchymal stem cell-induced doxorubicin resistance in triple negative breast cancer. Biomed Res Int. 2014; 2014: 532161 10.1155/2014/532161 25140317 PMC4124237

[17] Roodhart JM, Daenen LG, Stigter EC, Prins HJ, Gerrits J, Houthuijzen JM, et al Mesenchymal stem cells induce resistance to chemotherapy through the release of platinum-induced fatty acids. Cancer Cell. 2011 9 13; 20(3): 370–383. 10.1016/j.ccr.2011.08.010 21907927

[18] Schiff PB, Fant J, Horwitz SB. Promotion of microtubule assembly in vitro by taxol. Nature 1979 2 22; 227(5698): 665–7. 423966 10.1038/277665a0

[19] Zhao J, Kim JE, Reed E, Li QQ. Molecular mechanism of antitumor activity of taxanes in lung cancer (review). Int J Oncol. 2005 7; 27(1): 247–256. 15942666

[20] Wang Y, Chen Q, Jin S, Deng W, Li S, Tong Q, et al Up-regulation of P-glycoprotein is involved in the increased paclitaxel resistance in human esophageal cancer radioresistant cells. Scand J Gastroenterol. 2012 7; 47(7): 802–8. 10.3109/00365521.2012.683042 22545578

[21] Holton RA, Somoza C, Kim HB, Liang F, Biediger RJ, Boatman PB, et al First total synthesis of taxol. 1. Functionalization of the B ring. J. Am. Chem. Soc. 1994 116(4): 1597–1598.

[22] Holton RA, Kim HB, Somoza C, Liang F, Biediger RJ, Boatman PB, et al First iotal synthesis of Taxol. 2. Completion of the C and D Rings. J. Am. Chem. Soc. 1994 116(4): 1599–1600.

[23] Livak KJ, Schmittgen TD. Analyzing of relative gene expression data using real-time quantitative PCR and the 2^-ΔΔCt^ method. Methods 2001; 25(4): 402–408. 11846609 10.1006/meth.2001.1262

[24] Sekiya I, Larson BL, Vuoristo JT, Chi JG, Prockop DJ. Adipogenic differentiation of human adult stem cells from bone marrow stroma (MSCs). J Bone Miner Res. 2004 2; 19(2): 256–64. 14969395 10.1359/JBMR.0301220

[25] Del Sal G, Ruaro EM, Utrera R, Cole CN, Levine AJ, Schneider C. Gas1-induced growth suppression requires a transactivation-independent p53 function. Mol Cell Biol. 1995 12; 15(12): 7152–7160. 8524283 10.1128/mcb.15.12.7152 PMC230971

[26] Ishii M, Koike C, Igarashi A, Yamanaka K, Pan H, Higashi Y, et al Molecular markers distinguish bone marrow mesenchymal stem cells from fibroblasts. Biochem Biophys Res Commun. 2005 6 24; 332(1): 297–303. 15896330 10.1016/j.bbrc.2005.04.118

[27] Halfon S, Abramov N, Grinblat B, Ginis I. Markers distinguishing mesenchymal stem cells from fibroblasts are downregulated with passaging. Stem Cells Dev. 2011 1; 20(1): 53–66. 10.1089/scd.2010.0040 20528146

[28] Schiff PB, Horwitz SB. Taxol stabilizes microtubules in mouse fibroblast cells. Proc Natl Acad Sci U S A. 1980 3; 77(3): 1561–1565. 6103535 10.1073/pnas.77.3.1561 PMC348536

[29] Leonard GD, Fojo T, Bates SE. The role of ABC transporters in clinical practice. Oncologist. 2003; 8(5): 411–424. 14530494 10.1634/theoncologist.8-5-411

[30] Weaver BA. How Taxol/paclitaxel kills cancer cells. Mol Biol Cell. 2014 9 15; 25(18): 2677–81. 10.1091/mbc.E14-04-0916 25213191 PMC4161504

[31] Mueller LP, Luetzkendorf J, Mueller T, Reichelt K, Simon H, Schmoll HJ. Presence of mesenchymal stem cells in human bone marrow after exposure to chemotherapy: Evidence of resistance to apoptosis induction. Stem Cells. 2006 12; 24(12): 2753–2765. 16931776 10.1634/stemcells.2006-0108

[32] Polioudaki H, Kastrinaki MC, Papadaki HA, Theodoropoulos PA. Microtubule-interacting drugs induce moderate and reversible damage to human bone marrow mesenchymal stem cells. Cell Prolif. 2009 8; 42(4): 434–447. 10.1111/j.1365-2184.2009.00607.x 19486015 PMC6495966

[33] Duchi S, Dambruoso P, Martella E, Sotgiu G, Guerrini A, Lucarelli E, et al Thiophene-based compounds as fluorescent tags to study mesenchymal stem cell uptake and release of taxanes. Bioconjug Chem. 2014 4 16; 25(4): 649–655. 10.1021/bc5000498 24628247

[34] Straubinger RM, Sharma A, Murray M, Mayhew E. Novel Taxol formulations: Taxol-containing liposomes. Monogr Natl Cancer Inst. 1993; 15: 69–78. 7912532

[35] Pessina A, Bonomi A, Cocce V, Invernici G, Navone S, Cavicchini L, et al Mesenchymal stromal cells primed with paclitaxel provide a new approach for cancer therapy. PLoS One. 2011; 6(12): e28321 10.1371/journal.pone.0028321 22205945 PMC3243689

[36] Alt E, Yan Y, Gehmert S, Song YH, Altman A, Gehmert S,. et al Fibroblasts share mesenchymal phenotypes with stem cells, but lack their differentiation and colony-forming potential. Biol Cell. 2011 4; 103(4): 197–208. 10.1042/BC20100117 21332447

[37] Carcamo-Orive I, Tejados N, Delgado J, Gaztelumendi A, Otaegui D, Lang V, et al ERK2 protein regulates the proliferation of human mesenchymal stem cells without affecting their mobilization and differentiation potential. Exp Cell Res. 2008 5 1; 314(8): 1777–1788. 10.1016/j.yexcr.2008.01.020 18378228

[38] Lysy PA, Smets F, Sibille C, Najimi M, Sokal EM. Human skin fibroblasts: from mesodermal to hepatocyte-like differentiation. Hepatology. 2007; 46(5): 1574–1585. 17969047 10.1002/hep.21839

[39] Barbet R, Peiffer I, Hutchins JR, Hatzfeld A, Garrido E, Hatzfeld JA. Expression of the 49 human ATP binding cassette (ABC) genes in pluripotent embryonic stem cells and in early- and late-stage multipotent mesenchymal stem cells: Possible role of ABC plasma membrane transporters in maintaining human stem cell pluripotency. Cell Cycle. 2012 4 15; 11(8): 1611–1620. 10.4161/cc.20023 22456339

[40] Hall B, Dembinski J, Sasser AK, Studeny M, Andreeff M, Marini F. Mesenchymal stem cells in cancer: Tumor-associated fibroblasts and cell-based delivery vehicles. Int J Hematol. 2007 7; 86(1): 8–16. 17675260 10.1532/IJH97.06230

[41] Pessina A, Cocce V, Pascucci L, Bonomi A, Cavicchini L, Sisto F, et al Mesenchymal stromal cells primed with paclitaxel attract and kill leukaemia cells, inhibit angiogenesis and improve survival of leukaemia-bearing mice. Br J Haematol. 2013 3; 160(6): 766–778. 10.1111/bjh.12196 23293837

[42] Conforti A, Biagini S, Starc N, Proia A, Pessina A, Alessandri G, et al Human mesenchymal stromal cells primed with paclitaxel, apart from displaying anti-tumor activity, maintain their immune regulatory functions in vitro. Cytotherapy. 2014 6; 16(6): 868–870. 10.1016/j.jcyt.2014.01.414 24631286

[43] Moitra K, Lou H, Dean M. Multidrug efflux pumps and cancer stem cells: Insights into multidrug resistance and therapeutic development. Clin Pharmacol Ther. 2011 4; 89(4): 491–502. 10.1038/clpt.2011.14 21368752

[44] Takeda M, Mizokami A, Mamiya K, Li YQ, Zhang J, Keller ET, Namiki M. The establishment of two paclitaxel-resistant prostate cancer cell lines and the mechanisms of paclitaxel resistance with two cell lines. Prostate. 2007 6 15; 67(9): 955–67. 17440963 10.1002/pros.20581

[45] Guo B, Villeneuve DJ, Hembruff SL, Kirwan AF, Blais DE, Bonin M, Parissenti AM. Cross-resistance studies of isogenic drug-resistant breast tumor cell lines support recent clinical evidence suggesting that sensitivity to paclitaxel may be strongly compromised by prior doxorubicin exposure. Breast Cancer Res Treat. 2004 5; 85(1): 31–51. 15039596 10.1023/B:BREA.0000021046.29834.12

[46] Long BH, Fairchild CR. Paclitaxel inhibits progression of mitotic cells to G1phase by interference with spindle formation without affecting other microtubule functions during anaphase and telephase. Cancer Res. 1994; 15(54): 4355–61. 7913875

[47] Cheung TH, Rando TA. Molecular regulation of stem cell quiescence. Nat Rev Mol Cell Biol. 2013 6; 14(6): 329–340. 10.1038/nrm3591 23698583 PMC3808888

[48] Lee EA, Keutmann MK, Dowling ML, Harris E, Chan G, Kao GD. Inactivation of the mitotic checkpoint as a determinant of the efficacy of microtubule-targeted drugs in killing human cancer cells. Mol Cancer Ther. 2004 6; 3(6): 661–9. 15210851

[49] Touil Y, Igoudjil W, Corvaisier M, Dessein AF, Vandomme J, Monte D, et al Colon cancer cells escape 5FU chemotherapy-induced cell death by entering stemness and quiescence associated with the c-Yes/YAP axis. Clin Cancer Res. 2014 2 15; 20(4): 837–846. 10.1158/1078-0432.CCR-13-1854 24323901 PMC4387277

